# An Ovary Removed by Mistake for a Libial Cyst

**Published:** 1851-11

**Authors:** 


ARTICLE V.

*An Ovary removed by mistake for a Libial Cyst.*

At one of the late meetings of the Surgical Society of Paris, M GUERSANT, Chief Surgeon to the Hospital for Children, brought forward a case in which an error in diagnosis was committed, and which ended fatally. The patient was a little girl, eleven years of age, who, ever since she was one year old, had in the left labium a small painless tumor. Of late, however, this tumor had become troublesome, and interfered with walking. When examined, it was found of the size of a small walnut, situated in the thickness of the labium, and extremely movable, so much so, that it could be pushed downwards to the most posterior portion of the labium, and upwards as far as the external ring. It was, however, impossible to press the tumour into the ring, which latter presented no abnormal dilatation. The tumour had a great deal of analogy with a testicle. M. Guersant looked upon it as a cyst, and resolved to remove it. A longitudinal incision brought into view a membrane much resembling tunica vaginalis, and having the aspect of the peritoneum. Through this membrane an ovid body was observed, which was no other than the overy; it was attached to a pedicle formed by the Fallopian tube, which ran into the abdomen through the inguinal canal. M. Guersant placed a ligature on the pedicle, and cut out the ovary. Acute peritonitis occurred the very next day, and the patient died on the third day after the operation. M. Morel mentioned during the discussion that he had had an opportunity of seeing a tumour of the same kind in the labium, and formed by the ovary; no modification of size or sensibility was noticed to occur at the menstrual period. M. Lenoir stated that Pott has related a case in which the two ovaries were removed by an error in circumstances analogous to those of M. Guersant's patient.—*Prov. Med. and Surg. Jour., Aug. 6th*, 1851, *in Amer. Jour.*

